# Screening and Prognostic Analysis of Immune-Related Genes in Pancreatic Cancer

**DOI:** 10.3389/fgene.2021.721419

**Published:** 2021-10-19

**Authors:** Xin Wu, Yichao Liang

**Affiliations:** Department of General Surgery, Shengjing Hospital of China Medical University, Shenyang, China

**Keywords:** pancreatic cancer, CD19, immune cells within the tumor microenvironment, immunophenotyping, number of immune cells, immune infiltration score, consensus cluster analysis, survival analysis

## Abstract

Pancreatic cancer remains to have a high mortality, which is partly due to the lack of effective treatment strategies. In this study, genes with potential associations with immunophenotyping of pancreatic cancer were screened through bioinformatics analysis and the correlation between immune-related genes and the prognosis of pancreatic cancer patients was assessed. Firstly, differentially expressed immune genes were extracted from the pancreatic cancer-related datasets obtained for purposes of this study. The samples were processed by the “Consensus Cluster Plus” R package to determine the number of immune subtypes. Then, the pancreatic cancer immunophenotyping-related gene modules were determined. Differential analysis of immune gene modules was performed, and the function of genes related to pancreatic cancer immune subtypes was identified. The number of immune cells in the samples was calculated, followed by the differential expression analysis of immune cell numbers in each immune subtype of pancreatic cancer. The immune infiltration score was also estimated, and the correlation between the immune infiltration score and the patient prognosis with different immune subtypes was determined. Gene differences between each immune subtype were identified by differential expression analysis, and key immune genes affecting immunophenotyping were obtained. Following the analysis, 426 immune-related genes were identified to have potential involvement in the occurrence and development of pancreatic cancer, of which CD19 may be the most critical gene affecting the immunophenotyping of pancreatic cancer. CD19 played a significant role in the occurrence and development of IS2 and IS3 immune subtypes of pancreatic cancer through its action on B cells and T cells. Moreover, the expression of CD19 was increased in the collected pancreatic cancer tissues. Overall, our findings uncovered the critical role of CD19 in the prognosis of pancreatic cancer patients.

## Introduction

Pancreatic cancer is a malignancy known for its high mortality rates, with most patients receiving their diagnosis after the cancer has already reached an advanced stage; the risk factors consist of alcohol abuse, smoking, obesity, diabetes mellitus, family history, aging, and genetic factors (Zeng et al., 2019). Despite recent improvement in the standard of care for pancreatic cancer patients, their prognosis remains to be poor, with a 5-year survival rate of less than 5% (Li et al., 2019). Therefore, more sensitive and specific methods of diagnosis and treatment are essential to prolong the lives of pancreatic cancer patients.

A small proportion of pancreatic cancer patients with high effector T cell infiltration present with longer overall survival rate, indicating the potential for application of immunotherapy in the treatment of pancreatic cancer (Morrison et al., 2018). Immunotherapy uses the immune system to kill cancer cells, making it a safe and effective treatment option for numerous malignancies (Yang, 2015; Refolo et al., 2020). Cancer immunotherapy has been found to be effective in treatment of malignancies, including its recently revealed role in the treatments of lung cancer (Steven et al., 2016). The involvement of tumor microenvironment in tumorigenesis has been demonstrated since it influences the occurrence and progression of cancer by regulating the interaction between tumor cells and surrounding cells *via* the lymphatic and circulatory systems (Arneth, 2019). Moreover, the tumor microenvironment plays critical roles in therapeutic reaction and clinical result (Wu and Dai, 2017). More importantly, the immune cells within the tumor microenvironment significantly influence tumorigenesis, and these cells may fulfill antagonizing or promoting functions in tumors (Lei et al., 2020). Another report has indicated that immune system-associated genes have the potential to monitor the effectiveness of therapies and predict the prognosis of patients with liver cancer (Huang et al., 2020). Moreover, the infiltration of human tumors by lymphocytes has been studied, including its potential correlation with the prediction of prognosis (Sokratous et al., 2017). As a result, screening and prognostic analyses of immune-related genes in pancreatic cancer are essential to investigate the possible prognostic function of immune genes. This study was conducted with aims of identifying the immune-related genes in pancreatic cancer and their biological roles in prognosis of patient with this disease and other cancer immunotherapies.

## Materials and Methods

### The Cancer Genome Atlas (TCGA) Data Acquisition

The publicly available database TCGA (https://portal.gdc.cancer.gov/), The Genotype-Tissue Expression project (GTEx) (https://www.gtexportal.org/home/index.html), and Gene Expression Omnibus (GEO) (https://www.ncbi.nlm.nih.gov/gds) were employed to obtain pancreatic cancer related gene expression data, and the clinical information of samples was obtained from TCGA. In total, this study utilized PAAD (Pancreatic adenocarcinoma) dataset FPKM (FragmentsPer kilobase per Million) data in TCGA and six datasets in GEO.

In order to study cancer samples in TCGA, we obtained the data of 167 normal pancreatic samples from GTEx for pooling (PAAD^G^) because there were only four normal samples in PAAD. We calculated the number of samples with gene expression of PAAD^G^ as 0 and deleted 100 genes above 0 value (PAAD^D^). Then, the pancreatic cancer samples were extracted in PAAD^D^ and the clinical data was compared, the results of which yielded that one of the samples had no clinical information, therefore, the remaining 177 samples (PAAD^T^) were extracted. To further analyze immune-related genes in pancreatic cancer, 426 differentially expressed immune genes were obtained from PAAD^T^ (PAAD^TI^). The accuracy of immune subtypes was determined by merging six pancreatic cancer datasets with control and cancer groups obtained from the GEO database, which was named PCG once the batch effect was processed, the normal samples were deleted (PCG^T^), the PAAD^T^ dataset was aligned, and the subset of 303 immune genes (PCG^TI^) of PCG^T^ was extracted. The details of expression dataset of the genes are shown in Table 1, and the clinical information of TCGA^T^ is shown in Supplementary Table S1. In order to obtain immune gene names, we used The Immport database obtain immune gene names (https://www.Immport.org/shared/home), for which “Gene Summary” was obtained by first selecting the “Resources” tab, followed by the “Gene Lists” tab, and the immune-related genes were downloaded. Finally, a total of 1811 immune genes were obtained.

**TABLE 1:** Gene expression data.

Dataset	Platform	Samples, n (control/tumor)	GeneNum	Handle
PAAD		182 (4/178)	56,753	
PAAD^G^		349 (171/178)	54,751	
PAAD^D^		349 (171/178)	23,303	
PAAD^T^		177 (0/177)	23,303	
PAAD^TI^		177 ((0/177)	426	
PCG		170 (55/115)	16,494	
PCG^T^		115 (0/115)	16,494	
PCG^TI^		115 (0/115)	303	
GSE15932	GPL570	16 (8/8)	21,655	Log2 processing
GSE16515	GPL570	52 (16/36)	21,655	Log2 processing
GSE22780	GPL570	16 (8/8)	21,655	Log2 processing
GSE32676	GPL570	32 (7/25)	21,655	
GSE71989	GPL570	21 (8/13)	21,655	
GSE91035	GPL22763	33 (8/25)	21,189	

Note: Log2 processing, log2 transformation of data.

### Dataset Data Processing

Because of the large gene expression values for datasets GSE15932, GSE16515, and GSE22780, we log2-transformed their expression values using the R language. In order to eliminate batch effects between the six datasets of GEO, we first merged the expression data of the six datasets according to the genes. Since there were some differences in the data due to different sequencing platforms, the genes common to all datasets were retained during the merging process in order to ensure the accuracy of the data and used the “sva” software package (https://bioconductor.org/packages/sva/) to process the pooled data. To compare gene expression between each immune subtype of PAAD^TI^, the genes were normalized in units and the average gene expression was determined with immune subtype serving as the expression value of that gene in that immune subtype.

### Screening of Differentially Expressed Genes

We conducted differential expression analysis of the differential significance of expression of PAAD^D^ dataset genes between control and pancreatic cancer groups using the R language “limma” package (https://bioconductor.org/packages/limma/). A value of *p* < 0.05 was considered as statistically significant and used as the screening threshold for differentially expressed genes.

### Survival Analysis

According to the first 75% and the last 25% or the first 70% and the last 30% of the immune infiltration score, the patients were divided into high level of immune infiltration and low level of immune infiltration. Further correction of the significant *p* value obtained by the chi-square test was conducted via survival analysis using the R language “survival” software package (https://github.com/therneau/survival).

### Consensus Cluster Analysis

By the R language “Consensus Cluster Plus” package (https://bioconductor.org/packages/ConsensusClusterPlus/), consensus cluster analysis was conducted with a maximum number of clusters of 10, 50 repeats, a sample proportion of 0.8, and a ratio of features to samples of 1. The pam cluster algorithm was selected, and cluster analysis was performed using Pearson as a distance function. Cumulative distribution function (CDF) curve was drawn, where the number of possible clusters in the dataset was obtained using the unsupervised clustering method through the R language Consensus Cluster Plus package. The smooth curve indicates a reasonable clustering result. After the cluster analysis was completed, PCA analysis was performed using the “limma” package and classification dot plots were drawn to verify the cluster results.

### Pearson Correlation Analysis

Pearson correlation analysis was performed using R language, with *p* < 0.05 as a statistically significant correlation. The number of immune-related genes with significant gene correlation was counted, with the immune-related genes regarded as those with more than 300 related immune genes.

### Gene Ontology (GO) Enrichment Analysis

GO functional enrichment analysis was performed using the R language “cluster Profiler” package (https://guangchuangyu.github.io/software/clusterProfiler/), and *p* < 0.05 was considered statistically significant.

### Kyoto Encyclopedia of Genes and Genomes (KEGG) Enrichment Analysis

KEGG enrichment was performed using the database KOBAS 3.0 (KEGG-Orthology-Based Annotation System) (http://kobas.cbi.pku.edu.cn/kobas3), and *p* < 0.05 was considered statistically significant.

### Deconvolution Algorithm

The number of 22 different immune cells in 177 tumor samples was estimated using a deconvolution algorithm through the R language “e1071” software package (https://mirrors.sjtug.sjtu.edu.Cncran/package = e1071) and the “preprocess Core” package (https://bioconductor.org/packages/preprocessCore/). The *t*-test was used to compare the mean values of the number of immune cells between each immune subtype, and the significance level was set at 0.05 to determine whether there was a significant difference.

### Estimate Score of Immune Infiltration

Immune infiltration score was performed through the R language Estimate software package (https://r-forge.r-project.org/projects/Estimate/). Estimate package can be used to evaluate the Stromal Score, Immune Score, Estimate Score, and Tumor Purity of the samples through the gene expression. Pairwise comparison was performed for the mean immune infiltration score between each immune subtype using *t*-test. *p* < 0.05 was considered statistically significant.

### Construction of Protein-Protein Interaction (PPI) Network

PPI network data of immune genes were obtained from STRING (https://string-db.org/) website. The PPI network was visualized by the software Cytoscape (https://cytoscape.org/).

### Clinical Sample Collection

Pathologically confirmed 32 cases of pancreatic tissues were collected from patients who underwent Whipple resection in the pancreatic department of Shengjing Hospital of China Medical University between March 2017 to December 2019, with complete clinical pathological data and postoperative follow-up. Adjacent normal tissues (5 cm away from the tumor, confirmed by histopathological sections) were obtained from patients. None of the patients had received chemotherapy, radiotherapy, etc. prior to the surgery. The tissue specimens were obtained immediately after surgery. All patients submitted written informed consent and the human experiments were approved by the ethics committee of Shengjing Hospital of China Medical University. The study was guided by the intentions of the *Declaration of Helsinki* and ethical standard principles.

### Reverse Transcription Quantitative Polymerase Chain Reaction (RT-qPCR)

Total RNA from tissues were extracted from Trizol (Invitrogen, Carlsbad, CA, Unites States), and the integrity of total RNA was detected by gel electrophoresis. The total RNA was successfully extracted when 28S and 18S bands were clear and 28S band was about twice the 18S band. RNA concentration was measured at the absorbance of 260 and 280 nm using Nanodrop 2000 (Thermo Fisher, Austin, Texas, Unites States), and 1 μg of total RNA reversely transcribed into complementary DNA (cDNA) using Primescript™ RT Reagent Kit with GDNA Eraser Kit (4366596; Thermo Fisher). RT-qPCR experiments were carried out on the ABI7500 quantitative PCR instrument (Thermo Fisher) using SYBR^®^Premix Ex TaqTM (Tli RNaseH Plus) (RR820A, Takara, Tokyo, Japan). With glyceraldehyde-3-phosphate dehydrogenase (GAPDH), the relative expression level of miRNA was calculated using the 2^−ΔΔCt^ method. The primers (Supplementary Table S2) were provided by GenePharma (Shanghai, China). The experiment was conducted in triplicates.

### Western Blot Analysis

The total protein from tissues from extracted using Phenyl methylsulfonyl Fluoride (PMSF)-containing protease inhibitor (p0100, Solarbio, Beijing, China) in strict accordance with the instructions. The supernatant was extracted, and the protein concentration of each sample was determined by bicinchoninic acid (BCA) kit (23,227, Thermo Fisher). The protein was quantified according to different concentrations, transferred to polyvinylidene fluoride (PVDF) membrane by polyacrylamide gel electrophoresis, and sealed with 5% bovine serum albumin (BSA) at room temperature for 1 h. The membrane underwent incubation with primary antibodies against CD19 (ab134114; 1: 2000, Abcam, Cambridge, United Kingdom) and β-actin (ab8226; 1: 5,000, Abcam) overnight at 4 °C, and then secondary antibody horseradish peroxidase (HRP) labeled goat anti rabbit Immunoglobulin G (IgG; ab205718; 1: 20,000, Abcam) at room temperature for 1.5 h, followed by color development using developer (NCI4106, pierce, Rockford, IL, Unites States). ImageJ 1.48u software (Bio-rad, Hercules, CA, Unites States) was used for protein quantitative analysis, and the relative protein content was expressed by the gray value of the corresponding protein band and the gray value of β-actin. Each experiment was conducted in triplicates.

### Statistical Analysis

Data are shown as the mean ± standard deviation from at least three independent experiments. Statistical comparisons were performed using unpaired *t*-test when only two groups were compared, or by Tukey’s test-corrected one-way analysis of variance (ANOVA) when more than two groups were compared. All statistical analyses were completed with SPSS 21.0 software (IBM, Armonk, NY, Unites States) with *p* < 0.05 indicating statistical significance.

## Results

### A Total of 426 Immune-Related Genes May Be Involved in the Development and Progression of Pancreatic Cancer

**FIGURE 1:**
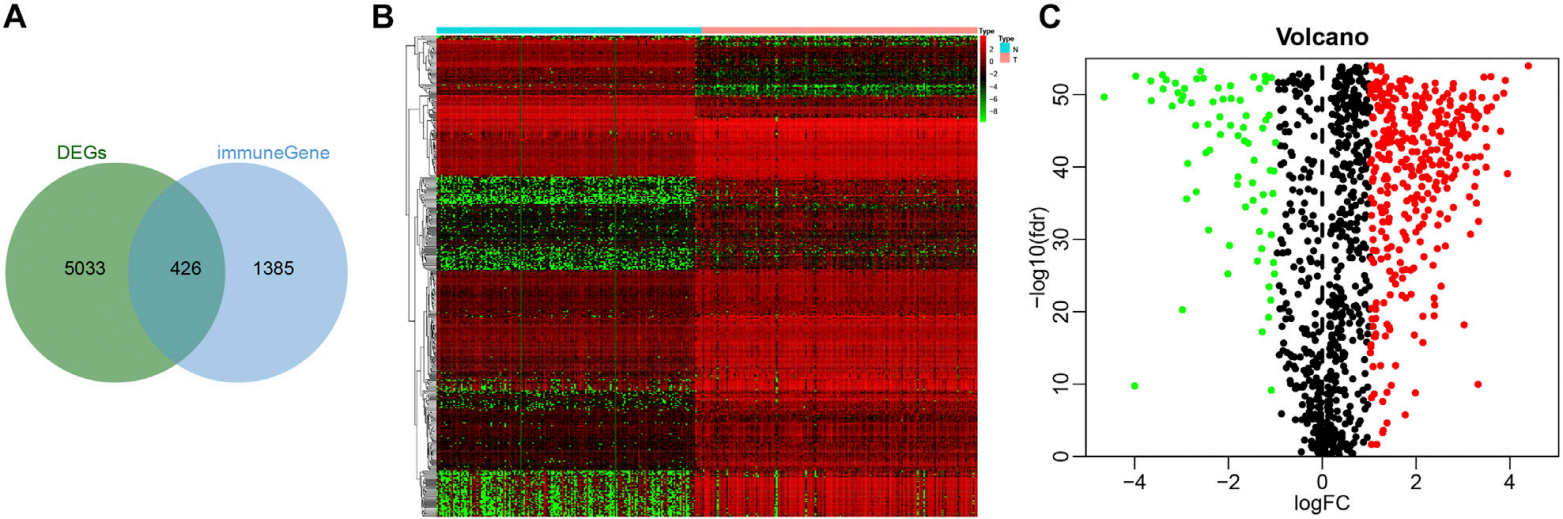
Screening of immune-related genes in pancreatic cancer samples. **(A)** Venn diagram showing the intersection of immune-related genes obtained by PAAD^D^ and Immport database (426 intersection genes). **(B)** Heatmap showing the expression of 426 intersection genes (color scale of gene expression in the upper right). **(C)** Volcano diagram showing the expression of 426 intersection genes (green dot indicates significantly down-regulated immune-related genes, red dot indicates significantly up-regulated immune-related genes, and black dot indicates immune-related genes with no significant difference).

To screen immune-related genes in pancreatic cancer, 5,459 differentially expressed immune-related genes were identified by analyzing the dataset PAAD^D^, including 2,827 genes with a high expression and 2,632 with a low expression. Moreover, 1811 human immune-related genes were obtained from the Immport database. Finally, the intersection was obtained, the results of which yielded 426 differentially expressed immune-related genes (Figure 1A), including 352 highly expressed immune genes and 74 lowly expressed immune genes (Figures 1B,C). These findings suggested that 426 differentially expressed immune-related genes may be involved in the incidence and development of pancreatic cancer.

### Pancreatic Cancer Samples Are Divided Into Four Different Immune Subtypes (IS1, IS2, IS2, and IS4 Types)

**FIGURE 2:**
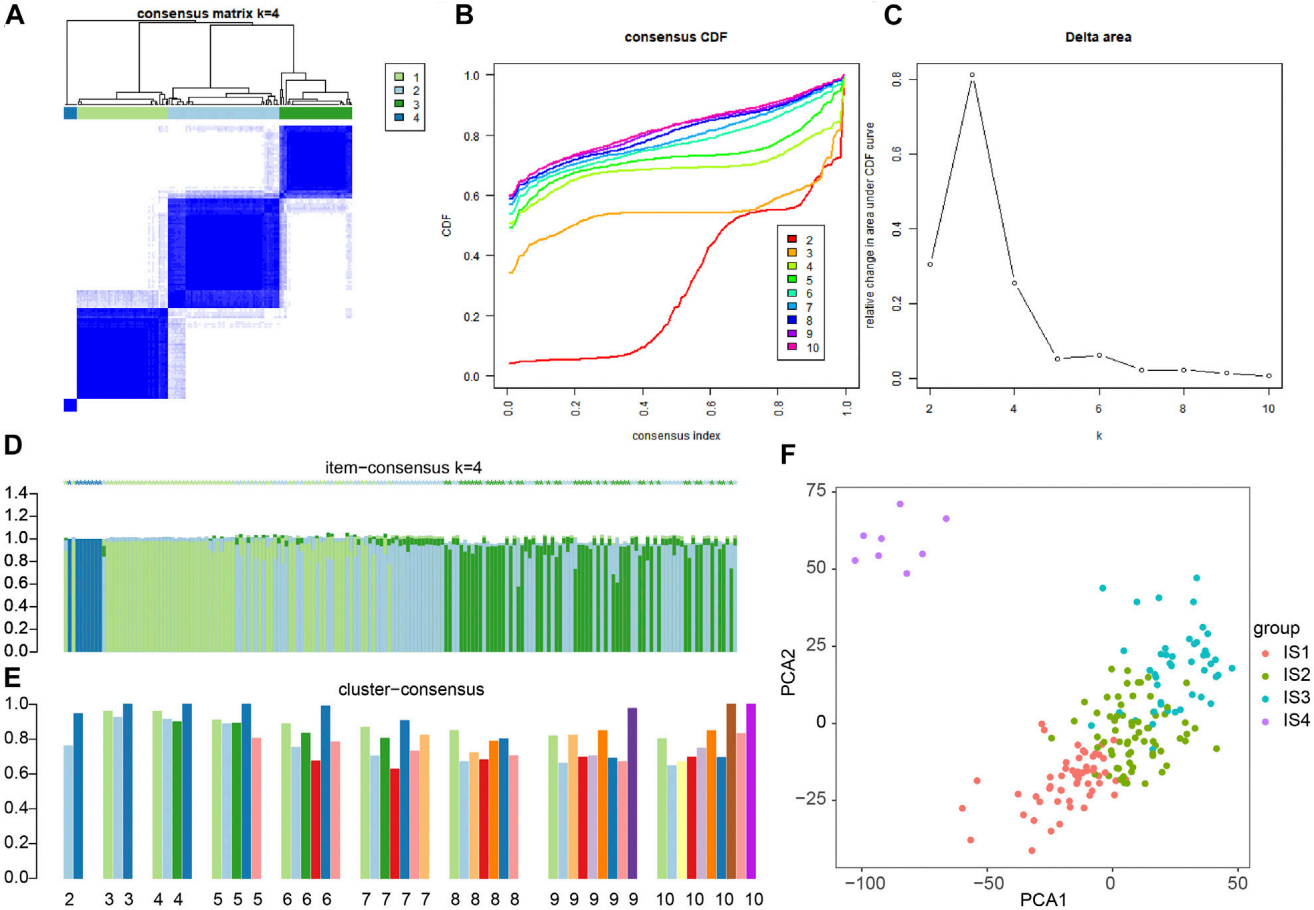
Consensus cluster analysis of differentially expressed gene datasets in pancreatic cancer samples to determine the number of immunophenotyping. **(A)** The cluster map at k = 4; the clearer for each category, the higher the consensus, the better the clustering result. **(B)** The consensus CDF curve at k = 2–10; the curve tends to be smooth after reaching a certain value, indicating that the clustering result is better (the first smoother classification is selected in this study). **(C)** The line graph of CDF area under curve at k = 2–10; the last curve area with greater change was between k = 4 and k = 5 (the number of clusters k = 4 was selected in this study). **(D)** The item-consensus diagram at k = 4; *x*-axis indicates different samples, *y*-axis indicates the consensus of sample classification; the higher the color purity of histogram of classification consensus of pancreatic cancer samples, the higher the classification consensus of pancreatic cancer samples, the more accurate the classification results of pancreatic cancer samples. **(E)** The consensus cluster diagram of different pancreatic cancer sample classification at a cluster number of 2–10. The higher the overall value, the more accurate the results. **(F)** PCA analysis verifying the accuracy of different immunophenotyping of pancreatic cancer samples, and different color points indicate different clusters of pancreatic cancer samples in consensus clusters.

To improve the accuracy of immunophenotyping for pancreatic cancer, 2,267 different genes with significant associations containing at least 300 of the 426 significantly differential immune genes were selected and an immune subtype analysis model for pancreatic cancer was established using 2,267 genes (Supplementary Table S3). Next, a new dataset was prepared with the combination of 2,267 genes and 426 immune-related genes, and the samples of this dataset were subjected to consensus cluster analysis according to the expression values of the genes. The results revealed that among the different number of clusters (k) from 1 to 10, when the number of clusters was 4, the consensus CDF curve was nearly smooth, the area under the CDF curve changed little with clear consensus matrix classification and item-consensus columnar band, which indicated that at k = 4, the consistency of various types reached the highest (Figures 2A–E and Supplementary Figure S1). The above result provided evidence supporting the classification of pancreatic cancer samples into four immune subtypes; IS1, IS2, IS2, and IS4 (Supplementary Table S4). The results of PCA analysis revealed that the four immune subtypes in pancreatic cancer samples had different central points (Figure 2F). The above findings indicated that the four immune subtypes of pancreatic cancer may have different biological characteristics.

### The 426 Immune-Related Genes Have Different Expression Trends in the Four Immune Subtypes of Pancreatic Cancer Samples

**FIGURE 3:**
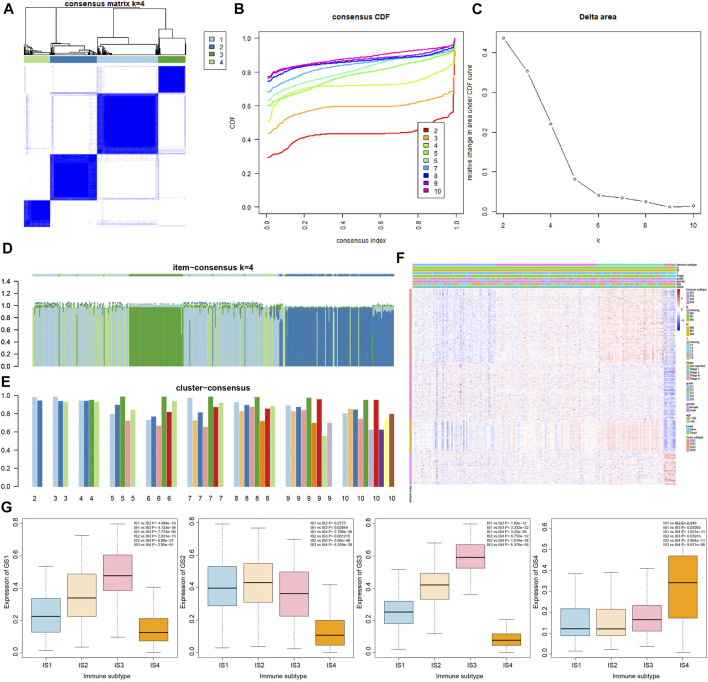
Correlation analysis of 426 immune-related genes with four immune subtypes of pancreatic cancer samples. **(A)** The cluster diagram at k = 4. The clearer the consensus is, the better the clustering result. **(B)** The consensus CDF curve at k = 2–10; the curve tends to be smooth after reaching a certain value, indicating that the clustering result is better (the first smoother classification was selected in this study). **(C)** The line graph of change in the area under the CDF curve at k = 2–10 (the number of clusters was selected as k = 4 in this study). **(D)** The item-consensus graph at k = 4, the *x*-axis indicates different immune-related genes, and the *y*-axis indicates the consistency of classification of 426 immune-related genes; the higher the color purity of the sample consensus histogram, the higher the consensus classification of 426 immune-related genes, and the more accurate the classification result. **(E)** Consensus cluster graph with the number of clusters of 2–10; the more accurate the overall value, the more accurate the results. **(F)** The expression heatmap of 426 immune-related genes in the sample, wherein the clinical attributes of the samples were also shown. **(G)** The overall expression trend of the four gene types of GS1, GS2, GS3, and GS4 in the four immune subtypes of pancreatic cancer samples, and the significance of difference between modules is shown in the upper right corner.

In order to determine the relationship between 426 immune-related genes and immune subtypes of pancreatic cancer samples, consensus cluster analysis was conducted again. The results revealed that when the number of clusters was 4 (the number of clusters was between 1 and 10), the consensus CDF curve was close to smooth, the area under the CDF curve changed little, with clear consensus matrix classification and the item-consensus columnar band (Figures 3A–E and Supplementary Figure S2). The above results showed that the most suitable cluster number was 4, and 426 genes can be classified into four categories: namely, GS1, GS2, GS3 and GS4 (Supplementary Table S5); the number of four categories of genes was 161, 123, 72 and 70, respectively.

The results of heatmap analysis showed that the four types of genes had different expression trends in the four immune subtypes of pancreatic cancer samples (Figure 3F). As shown in Figure 3G, the overall expression of GS1 gene and GS3 gene in the four immune subtypes of pancreatic cancer samples was observed to have a significant difference following procedures involving normalizing the data and then plotting the boxplot, and the expression trends were IS3 > IS2 > IS1 > IS4. In addition, the expression trends of GS2 gene in the four immune subtypes were IS2 ≈ IS1 > IS3 > IS4, and the expression trends of GS4 gene were IS4 > IS3 > IS1 ≈ IS2.

### The Four Classes of Immune-Related Genes Play Different Biological Functions in Samples of the Four Immune Subtypes of Pancreatic Cancer

**FIGURE 4:**
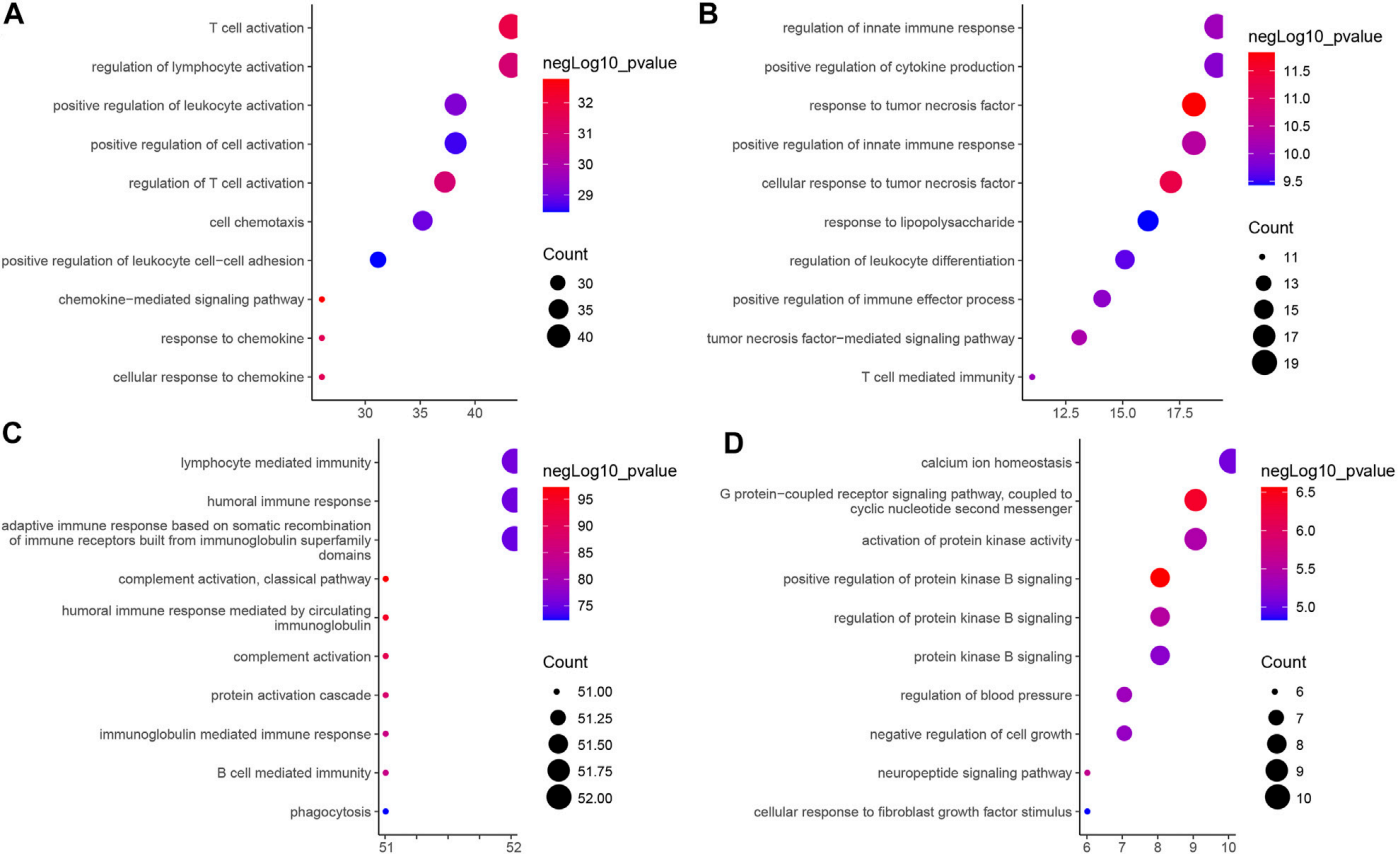
GO functional enrichment analysis of four types of immune-related genes in four immune subtype samples of pancreatic cancer. **(A)** GS1. **(B)** GS2. **(C)** GS3. **(D)** GS4. The vertical axis indicates the enriched GO functional item, the horizontal axis and the size of bubbles indicate the number of genes enriched in the item, and the color of bubble indicates the enrichment significance -lgP value.

As shown in Figure 4, we further performed GO functional enrichment analysis on the four types of immune-related genes, and found that GS1, GS2, GS3 and GS4 may be related to chemokines, tumor necrosis factor, immunoglobulins, and protein kinases, respectively. Combined with the results of consensus cluster analysis, we speculated that pancreatic cancer IS1 immune subtype and IS2 immune subtype may be associated with tumor necrosis factor and protein kinase, whose main function may be to regulate the development of pancreatic cancer cells; IS3 immune subtype had the highest expression of GS1 gene and GS3 gene, which may be a new immune subtype of pancreatic cancer; IS4 immune subtype had the lowest expression of GS1 gene, GS2 gene, and GS3 gene and the highest GS4 level, which may be a subtype related to protein kinase. Taken together, the expression trends of the four types of immune-related genes in the four immune subtype samples of pancreatic cancer were different, and the biological functions exerted by them in pancreatic cancer were also significantly different.

### The Four Immune Subtypes of Pancreatic Cancer Samples Show a Clear Difference in the Number of Immune Cells

**FIGURE 5:**
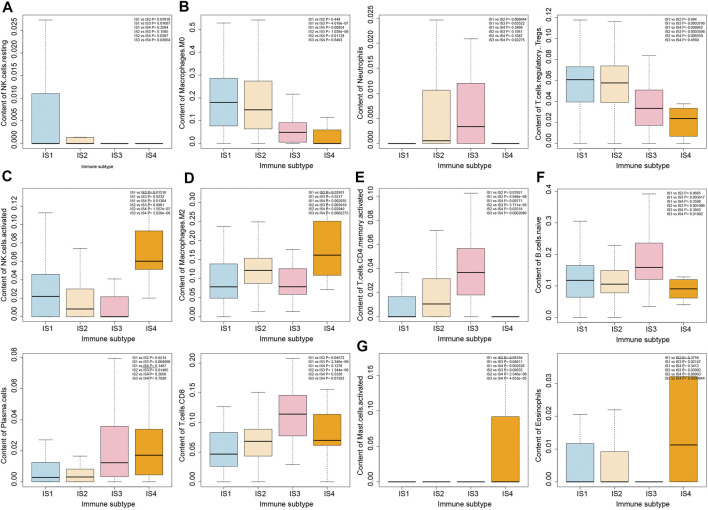
Differential analysis of immune cell number in four immune subtypes of pancreatic cancer samples. **(A)** Box plot of content of NK cells in four immune subtypes. **(B)** Box plot of content of Macrophages M0, Neutrophils, and T cells regulatory Tregs in four immune subtypes. **(C)** Box plot of content of NK cells activated in four immune subtypes. **(D)** Box plot of content of Macrophages M2 in four immune subtypes. **(E)** Box plot of T cells CD4 memory activated in four immune subtypes. **(F)** Box plot of content of B cells native, Plasma cells and T cells CD8 in four immune subtypes. **(G)** Box plot of content of Mast cells activated and Eosinophils in four immune subtypes. The significance of difference is shown in the upper right corner of the image.

To further analyze the biological characteristics of the four immune subtypes of pancreatic cancer samples, we estimated the number of 22 types of immune cells in the four immune subtypes of pancreatic cancer samples by the deconvolution algorithm (Supplementary Figure S3). As shown in Figure 5, more NK cell resting was observed in IS1 immune subtype pancreatic cancer samples, more Neutrophils, Macrophages M0 and T cells regulatory Tregs in IS1 immune subtype and IS2 immune subtype pancreatic cancer samples, more NK cells activated in IS1 immune subtype and IS4 immune subtype pancreatic cancer samples, more Macrophages M2 in IS2 immune subtype and IS4 immune subtype pancreatic cancer samples, more T cells CD4 memory cells in IS2 immune subtype and IS3 immune subtype pancreatic cancer samples, more B cells, Plasma cells, and T cells CD8 in IS3 immune subtype pancreatic cancer samples, and more Mast activated cells and Eosinophils in IS4 immune subtype pancreatic cancer samples.

### The Immune Infiltration Scores of the Four Immune Subtypes of Pancreatic Cancer Samples Are Closely Correlated With the Number of Immune Cells

**FIGURE 6:**
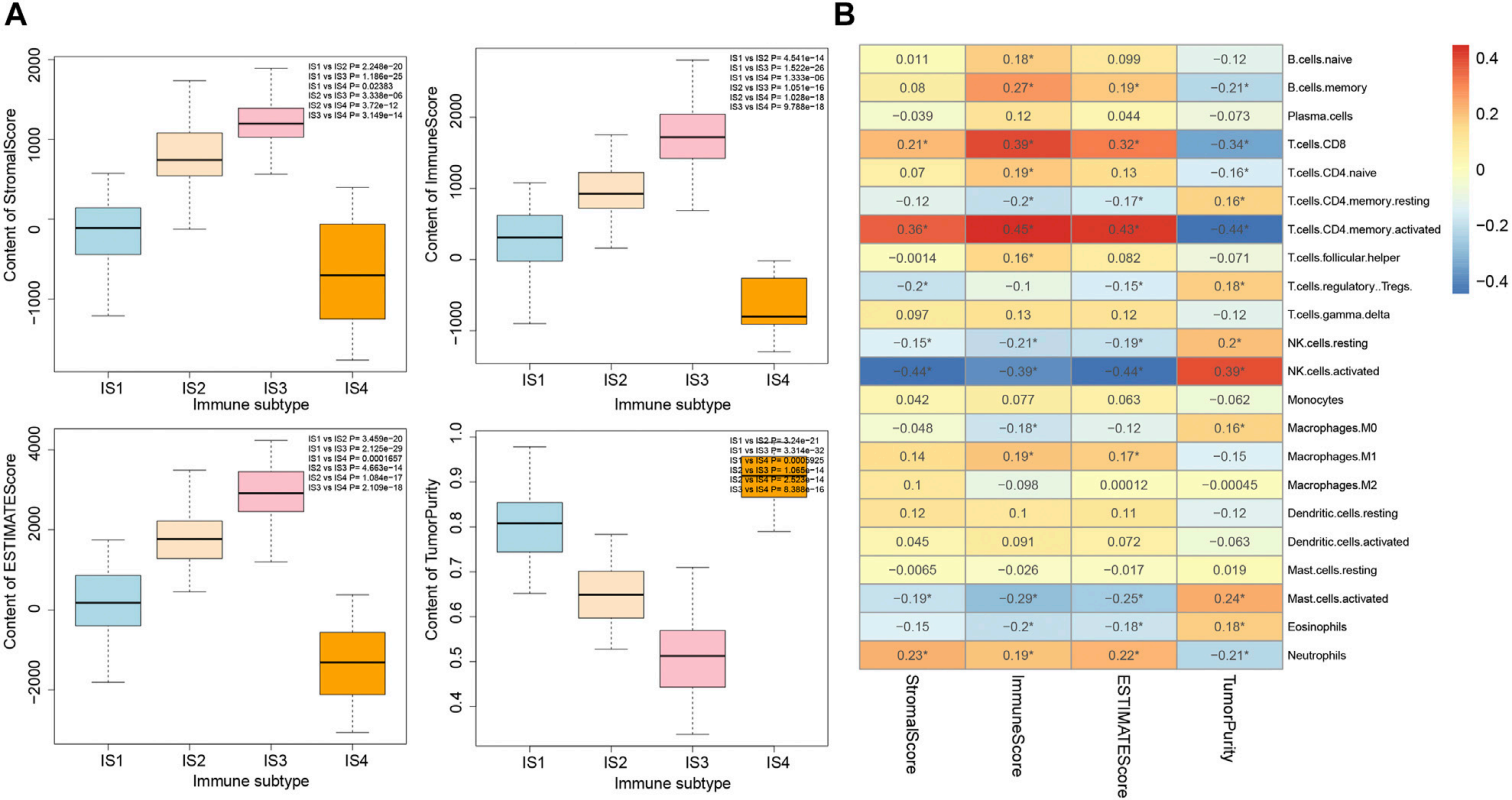
Correlation analysis between immune infiltration score and immune cell number in four immune subtypes of pancreatic cancer samples. **(A)** Box plot for the comparison of immune infiltration scores of the four immune subtypes of pancreatic cancer samples (the significance of difference is shown in the upper right corner). **(B)** Heatmap of the correlation between immune infiltration scores of the four immune subtypes of pancreatic cancer samples and the number of immune cells; *p < 0.05.

It was found by estimating the immune infiltration scores of the four immune subtypes of pancreatic cancer samples (Figure 6A) that the Stromal Score, Immune Score, and Estimate Score of the four immune subtypes of pancreatic cancer samples were significantly different, and their trends were IS3 > IS2 > IS1 > IS4. In addition, the Tumor Purity of the four immune subtypes of pancreatic cancer samples were also significantly different, and their trends were IS4 > IS1 > IS2 > IS3. The above results showed that there were significant differences in the immune infiltration scores among the four immune subtypes of pancreatic cancer samples.

The correlation between the number of immune cells and immune infiltration score in the four immune subtypes of pancreatic cancer samples was analyzed, the results of which revealed that T cells CD8, T cells CD4 memory activated, NK cell resting, NK cell activated, Mast cell activated, and Neutrophils were significantly correlated with immune infiltration score (Figure 6B). Therefore, we speculated that the occurrence of IS1 immune subtype pancreatic cancer may be related to NK cell resting and NK cell activation, the occurrence of IS2 immune subtype pancreatic cancer may be related to Neutrophils and T cells CD4 memory activation, the occurrence of IS3 immune subtype pancreatic cancer may be related to T cells CD4 memory activation and T cells CD8 activation, while the occurrence of IS4 immune subtype pancreatic cancer may be related to protein kinase.

### The Immune Infiltration Score of the Four Immune Subtypes of Pancreatic Cancer Samples Is Closely Related to Patient Prognosis

**FIGURE 7:**
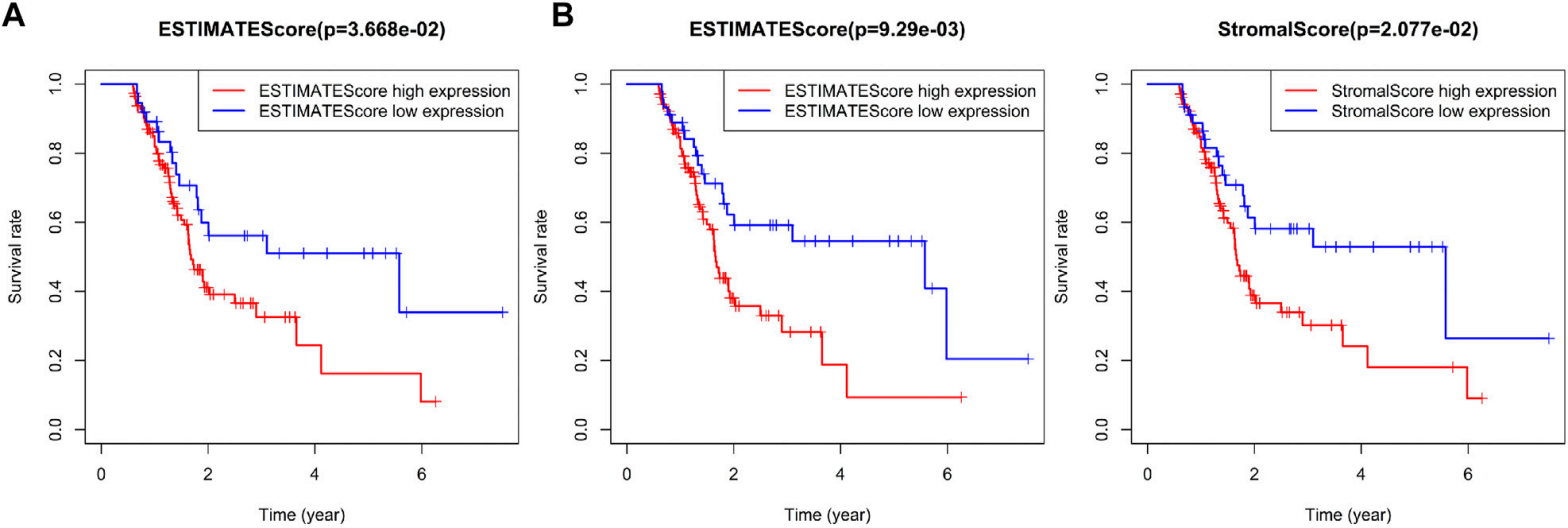
Correlation analysis between prognosis and immune infiltration score in patients with four immune subtypes of pancreatic cancer. **(A)** Survival difference between 75% of pancreatic cancer samples with high immune invasion score and 25% of pancreatic cancer samples with low immune invasion score. **(B)** Survival difference between 70% of pancreatic cancer samples with high immune invasion score and 30% of pancreatic cancer samples with low immune invasion score.

The relationship between the immune infiltration score of the four immune subtypes of pancreatic cancer samples and the prognosis of patients by survival analysis was evaluated. First, the data of pancreatic cancer patients with survival time less than 200 was removed. Among the remaining 149 pancreatic cancer patients, the samples with Stromal Score, Immune Score, and Estimate Score of less than 0 in some pancreatic cancer patients were also removed, and their proportions were 24.1, 15.4, and 17.4%, respectively. We defined the samples with score in the first 75% as pancreatic cancer samples with high immune invasion score and the last 25% as pancreatic cancer samples with low immune invasion score. The results of survival analysis showed that the prognosis of pancreatic cancer patients with high immune infiltration score was relatively poor, while the prognosis of pancreatic cancer patients with low immune infiltration score was better (Figure 7A).

To further compare the prognostic difference between pancreatic cancer samples with high or low immune infiltration scores, the proportion of samples with high or low immune infiltration scores was adjusted to 70 and 30%, respectively. Secondary survival analysis also showed that high immune infiltration score predicted poor prognosis in patients with pancreatic cancer, while low immune infiltration score predicted good prognosis (Figure 7B). As shown in Supplementary Table S6, we found very few IS2 immune subtype and IS3 immune subtype in pancreatic cancer samples with low immune infiltration score, while most of IS1 immune subtype and almost all IS4 immune subtype pancreatic cancer samples belonged to low immune infiltration score. The above results showed that the immune infiltration score was closely related to the prognosis of patients with pancreatic cancer, and also highlighted the presence of significant differences in the prognosis of patients with the four immune subtypes of pancreatic cancer.

### CD19 May Be the Most Critical Gene Affecting Immunophenotyping in Pancreatic Cancer

**FIGURE 8:**
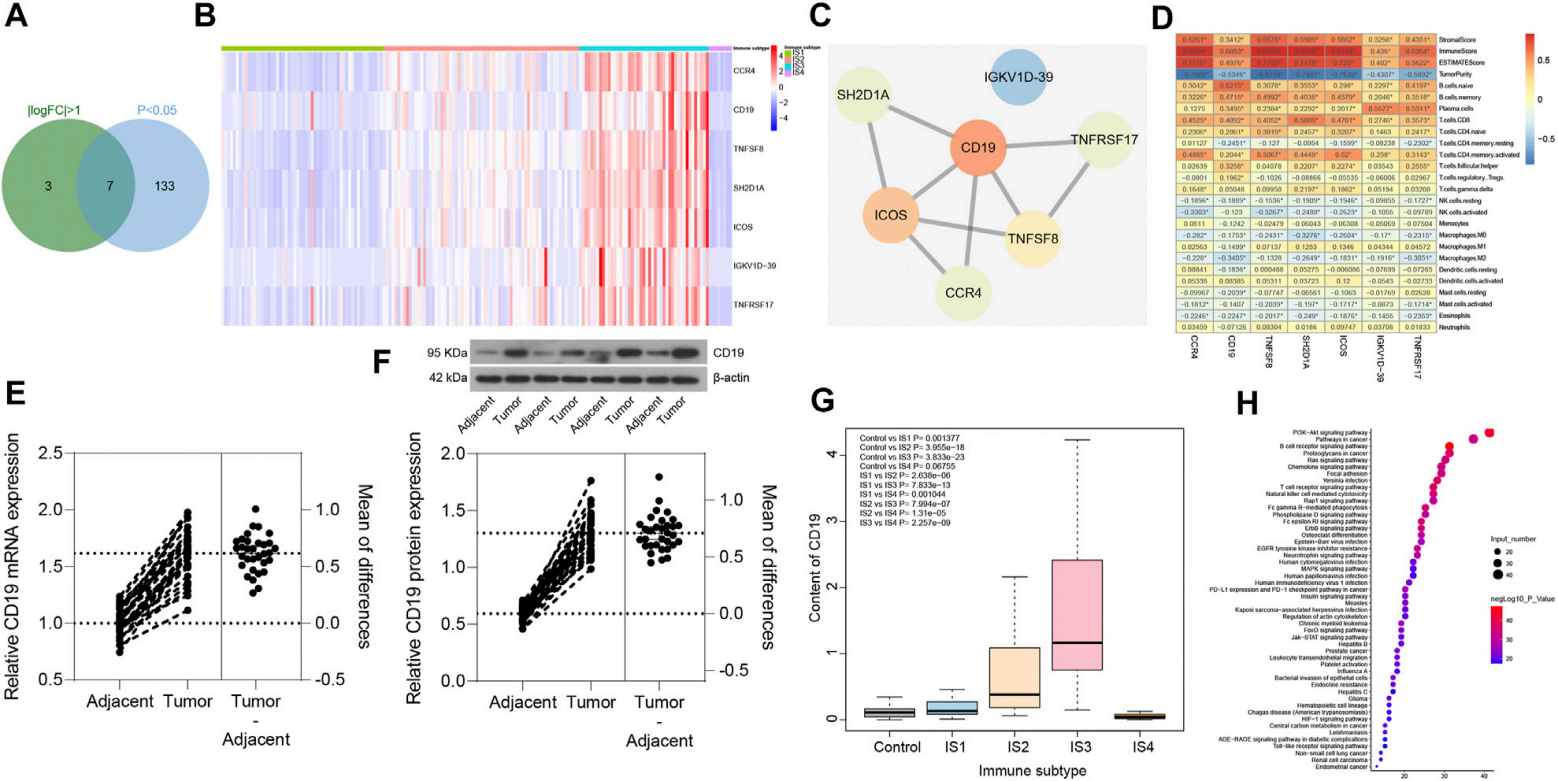
Screening of key immune genes in the four immune subtypes of pancreatic cancer samples. **(A)** Intersection of immune-related genes meeting 丨logFC丨 >1 and *p* < 0.05 in four immune subtypes of pancreatic cancer samples, and a total of seven intersection immune genes (CCR4, CD19, TNFSF8, SH2D1A, ICOS, IGKV1D-39, and TNFRSF17) were obtained. **(B)** Expression heatmap of seven intersection immune genes in four immune subtypes of pancreatic cancer samples. **(C)** PPI of seven intersection immune genes constructed by String database (the redder the gene is, the higher the core degree of the gene is; the bluer the gene is, the lower the core degree is; and the CD19 core degree is the highest in the network). **(D)** Correlation heatmap of the seven intersecting immune gene expression with immune infiltration score and immune cell number of pancreatic cancer samples (the square in the figure indicates the correlation strength, *p < 0.05). **(E)** the mRNA expression of CD19 in pancreatic cancer tissues and adjacent normal tissues determined with RT-qPCR (*p < 0.05 vs. adjacent normal tissues; n = 32). **(F)** the protein expression of CD19 in pancreatic cancer tissues and adjacent normal tissues determined with Western blot analysis (*p < 0.05 vs. adjacent normal tissues; n = 32). **(G)** Box plot of CD19 expression levels in the four immune subtypes of pancreatic cancer samples compared with normal samples (the significant difference is in the upper left corner). **(H)** Bubble diagram of the top 50 enriched signal pathways by KEGG analysis (the vertical axis indicates the enriched pathway, the horizontal axis indicates the number of genes enriched in this pathway, and the bubble color is shown in the right color scale; the redder the color, the higher the significance). The experiment was repeated three times independently.

In order to screen the key immune genes in the four immune subtypes of pancreatic cancer samples, differential expression analysis was performed on the 426 immune-related genes in the four immune subtypes of pancreatic cancer samples (丨logFC丨 >1 and *p* < 0.05). The results (Figures 8A,B) revealed seven immune-related genes (CCR4, CD19, TNFSF8, SH2D1A, ICOS, IGKV1D-39, and TNFRSF17) could potentially influence the immunophenotyping of pancreatic cancer. Subsequently, PPI was constructed between the seven immune-related genes through the String database, the findings of which showed that CD19 was the gene with the highest degree of core in the PPI network (Figure 8C). In addition, analysis of the relationship between the seven immune-related genes and the number of immune cells and immune infiltration score was conducted in pancreatic cancer samples. The results revealed that all the seven immune-related genes were closely related to the number of immune cells and immune infiltration score, and the number of immune cells related to CD19 expression was the most (Figure 8D). Moreover, we collected 32 cases of pancreatic cancer tissues and adjacent normal tissues, after which RT-qPCR and Western blot analysis were conducted, the results of which revealed that the expression of CD19 was increased in the pancreatic cancer tissues relative to adjacent normal tissues (Figures 8E,F). Based on this result, CD19 might be the most critical gene affecting immunophenotyping of pancreatic cancer.

The expression levels of CD19 was further assessed in the four immune subtypes of pancreatic cancer samples and normal samples, and was found (Figure 8G) to be upregulated in pancreatic cancer samples of IS1 immune subtype, IS2 immune subtype, and IS3 immune subtype compared with normal samples, while there was no significant difference in the expression between pancreatic cancer samples of IS4 immune subtype and normal samples. Previous findings confirmed that patients with pancreatic cancer of IS2 and IS3 immune subtypes have a poor prognosis (Figures 7A,B), which led to the understanding that upregulated CD19 expression could potentially predict a poor prognosis in patients with pancreatic cancer.

A total of 165 interacting genes of CD19 were obtained from the GeneCards database, and 156 significantly enriched signaling pathways associated with 165 genes were obtained by KEGG analysis. After careful analysis, it was found that B cell receptor signaling pathway and T cell receptor signaling pathway ranked first and fourth in the enrichment significance, respectively (Figure 8H), suggesting that CD19 might be involved in the occurrence and development of pancreatic cancer through B cells and T cells. The above results demonstrated that CD19 may be the most critical gene affecting the immunophenotyping of pancreatic cancer, and CD19 mainly affected the occurrence and development of IS2 and IS3 immune subtypes of pancreatic cancer through B cells and T cells.

### Reliability Analysis

**FIGURE 9:**
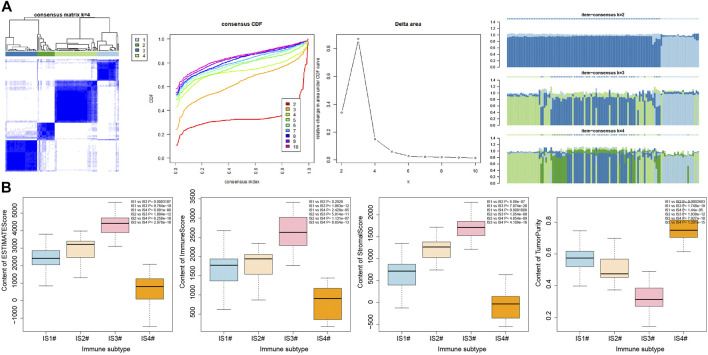
Secondary validation of six pancreatic cancer-related microarray datasets from GEO database. **(A)** Consensus cluster analysis results of six pancreatic cancer-related microarray datasets under the same model (the remaining cluster results are shown in Supplementary Figure 4). **(B)** Box plot of immune invasion score after classification (the significance of difference is shown in the upper right corner).

In order to verify the reliability of our results, six pancreatic cancer-related microarray datasets were retrieved using the GEO database, and 1784 genes for pancreatic cancer immunophenotyping were extracted after data merging and processing. Similarly, immunophenotyping of pancreatic cancer samples by consensus cluster analysis also showed (Figure 9A and Supplementary Figure S4) that clustering effect was most desirable when the number of clusters was 4. Through further analysis of the immune infiltration scores of the four immune subtypes of pancreatic cancer samples by Estimate, findings similar to PAAD^TI^ were observed (Figure 9B), providing further proof of the accuracy and reliability of this study.

## Discussion

Pancreatic cancer is predicted to surpass breast, colorectal, and prostate cancers to be the second leading cause of cancer-related mortality by 2030 (Li et al., 2019). The high mortality in these patients stems from the absence of clinical symptoms in most patients during the early stages of the disease and the low sensitivity and specificity of CA19-9 and other currently available biomarkers for pancreatic cancer (Zeng et al., 2019). Hence, there is an urgency in the need to explore novel diagnostic and prognostic biomarkers. In this paper, 426 immune-related genes were found to be involved in the occurrence and development of pancreatic cancer through bioinformatic analysis, of which CD19 may be the most critical gene affecting the immunophenotyping of pancreatic cancer. When compared to adjacent normal tissues, pancreatic cancer tissues were found to have upregulated CD19 expression.

Our analyses revealed that the immune-related genes CCR4, CD19, TNFSF8, SH2D1A, ICOS, IGKV1D-39, and TNFRSF17 may be implicated in the immunophenotyping of pancreatic cancer. An important reason for the high mortality observed in pancreatic cancer is its acquired immune privilege, which is stimulated by a small mutational burden, poor infiltration of T cells, and an immunosuppressive microenvironment (Morrison et al., 2018). As immunotherapy contributes to the innovation of clinical cancer management, ICOS is indicative of immune response mediated by T cells; therefore, ICOS ImmunoPET is recognized as a potential strategy to monitor, compare, and forecast successful application of immunotherapy in tumor (Xiao et al., 2020). More importantly, the growth of pancreatic cancer cells can be controlled by mesothelin virus-like particle immunization c via induction or reduction of CD8^+^ T cells in the frequency of CD4^+^ foxp3^+^ ICOS^−^ regulatory T cells (Zhang et al., 2013). CCR4, which is the receptor for two CC chemokine ligands of CCL17 and CCL22, may be applied in diseases concerning T-cell subsets in the clinic, thus attracting widespread attention (Yoshie and Matsushima, 2015). It has been also confirmed that up-regulated expression of CCR4^+^CD8^+^ T cells could inhibit the liver metastasis of pancreatic tumor cells (Zhan et al., 2017). Moreover, the blockade of CCR4 results in the reduction of regulatory T cells, while increasing the survival of canine models of bladder cancer (Maeda et al., 2019), and CCR4-CCL17/22 could serve as a chemokine gradient to chemoattract regulatory T cells to the tumor microenvironment (Ohue and Nishikawa, 2019). TNFSF8 is a member of the cytokines CD30 ligand/tumor necrosis factor superfamily and may serve as predictive biomarkers for pancreatic cancer patients' response to gemcitabine and erlotinib (Torres et al., 2014). In addition, TNFSF8 is more predictive for anti-PD-1 response as well as overall survival and progression-free survival when compared to programmed cell death protein ligand 1 (Cho et al., 2020). SH2D1A is an X-linked SLAM-related protein that is mainly expressed in NK, T, and several B cells, which is able to regulate humoral autoimmunity in a T-dependent manner (Hron et al., 2004). Besides, SH2D1A may be involved in the modulation of B-cell differentiation through switching signaling pathways mediated by CD150 (Mikhalap et al., 2004). In addition, TNFRSF17, also known as B cell maturation antigen, can induce breast cancer cell stemness mediated by B cell activating factor and a proliferation-inducing ligand (Pelekanou et al., 2018). It is worth noting that TNFRSF17 can serve as a biomarker in patients with systemic lupus erythematosus (Salazar-Camarena et al., 2020).

CD19 was identified as the most important gene among the seven immune-related genes that influences immunophenotyping of pancreatic cancer due to the finding that the number of immune cells related to CD19 expression were the most significant. Intriguingly, CD19 chimeric antigen receptor T-cell therapy possesses high effectiveness when treating B-cell lymphoma or B-cell acute lymphoblastic leukemia, which provides patients who had no response to conventional therapy with an alternative therapeutic option, and it can greatly improve patient prognosis, which makes it a promising therapeutic option (Xu et al., 2019). Moreover, it has been reported that T cells with chimeric antigen receptors have the potential to treat numerous types of cancers by targeting CD19, and down-regulation of CD19 expression by T cells with chimeric antigen receptors contributes to elimination of B cells, thereby impeding development and progression of pancreatic cancer (Morello et al., 2016; Chen et al., 2018; Sun et al., 2018; He et al., 2020; Ko et al., 2020). Although the role of CD19^+^CD24^+^CD38^+^ and CD19^+^CD24^+^CD27^+^ regulatory B cells have been investigated in patients with type 1 autoimmune pancreatitis (Sumimoto et al., 2014), the specific mechanism of action of CD19 is yet to be determined in pancreatic cancer. Moreover, the expression of CD19 has only been validated in human pancreatic adenocarcinoma patient samples obtained from TCGA dataset (Ko et al., 2020). This study found upregulated CD19 expression in the pancreatic cancer tissues in comparison to adjacent normal tissues. These findings suggest that high CD19 expression could predict poor prognosis in pancreatic cancer patients.

This study is the first of its kind, as it provides the first insight into immune-related genes with the capacity of predicting the prognosis of patients with pancreatic cancer, which provides a new theoretical basis regarding the role of immune cells within the tumor microenvironment in the development and progression of pancreatic cancer, thereby further elucidating the underlying therapeutic mechanism of immunotherapy methods. However, more studies still required to validate the accuracy of the present study.

## Data Availability

The datasets presented in this study can be found in online repositories. The names of the repository/repositories and accession number(s) can be found in the article/Supplementary Material.

## Supplementary Material

Supplementary Figure S1 Consensus cluster results based on samples at k = 1 to 10. **(A)** Block diagram of clusters from at k = 1 to 10. **(B)** Consensus CDF curve at k = 2 to 10. **(C)** Line diagram of change in area under CDF curve at k = 2 to 10. **(D)** Item-consensus diagram at k = 2 to 10. E, Consensus diagram of different clusters in the number of clusters 2-10.

Supplementary Figure S2 Gene-based consensus clustering results for cluster number k from 1 to 10. **(A)** Block diagram of clusters at k = 1 to 10. **(B)** Consensus CDF curve at k = 2 to 10. **(C)** Line diagram of change in area under CDF curve at k = 2 to 10. **(D)** Item-consensus diagram at k = 2 to 10. **(E)** Consensus diagram of different clusters in the number of clusters 2-10.

Supplementary Figure S3 Box plots of 22 different immune cell numbers in the four immune subtypes. Significance of difference is shown in the upper right corner.

Supplementary Figure S4 Consensus cluster results based on GEO database samples with cluster number k from 1 to 10. **(A)** Block diagram of clusters at k = 1 to 10. **(B)** Consensus CDF curve at k = 2 to 10. **(C)** Line diagram of change in area under CDF curve at k = 2 to 10. **(D)** Item-consensus diagram at k = 2 to 10. **(E)** Consensus diagram of different clusters in the number of clusters 2-10.

Supplementary Table S1 Clinical information of PADDT

Supplementary Table S2 Primer sequences for RT-qPCR

Supplementary Table S3 Correlation between genes and immune genes in pancreatic cancer

Supplementary Table S4 Immune subtypes of pancreatic cancer samples

Supplementary Table S5 clustering of immune gene subtypes

Supplementary Table S6 Distribution of immune subtypes in pancreatic cancer samples with high immune infiltration score and pancreatic cancer samples with low immune infiltration score
