# Supplementary material for: Screening and Prognostic Analysis of Immune-Related Genes in Pancreatic Cancer
Source: Front Genet. 2021 Oct 19;12:721419. doi: 10.3389/fgene.2021.721419 (PMC8560963; doi:10.3389/fgene.2021.721419)
Supplement: Supplementary file 1 [file DataSheet1.zip › Supplementary Tables/Table S1.docx]

**Table S1**Clinical information of PADD^T^

| TCGA discovery cohorts | | | |
| --- | --- | --- | --- |
| Dataset | PAAD^T^ | Gender, Female (%) | 45(80) |
| Tumor Number | 177 | T (%) |  |
| Median age (IQR) | 65(35-88) | T1 | 4.0(7) |
| Stage (%) |  | T2 | 13.5(24) |
| I | 11.9(21) | T3 | 79.7(141) |
| II | 81.9(145) | T4 | 1.7(3) |
| III | 1.7(3) | NA/TX | 1.1(2) |
| IV | 2.8(5) | M (%) |  |
| NA | 1.7(3) | M0 | 44.6(79) |
| Grade (%) |  | M1 | 2.8(5) |
| G1 | 17.0(30) | NA/MX | 52.6(93) |
| G2 | 53.7(95) | N (%) |  |
| G3 | 27.1(48) | N1 | 28.3(50) |
| G4 | 1.1(2) | N0 | 68.9(122) |
| NA/GX | 1.1(2) | NA | 2.8(5) |

Note: NA/GX/TX/MX indicates that the item cannot be assessed or data is missing.
