# Supplementary material for: Screening and Prognostic Analysis of Immune-Related Genes in Pancreatic Cancer
Source: Front Genet. 2021 Oct 19;12:721419. doi: 10.3389/fgene.2021.721419 (PMC8560963; doi:10.3389/fgene.2021.721419)
Supplement: Supplementary file 1 [file DataSheet1.zip › Supplementary Tables/Table S2.docx]

**Table S2** Primer sequences for RT-qPCR

| Gene | primer sequences |
| --- | --- |
| CD19 | F: 5'-GTCCCCGCTTAAACCCTTCT-3' |
|  | R: 5'-GGTTTCCATAAGACGGGGCA-3' |
| GAPDH | F: 5'-AATCCCATCACCATCTTCCA-3' |
|  | R: 5'-TGGACTCCACGACGTACTCA-3'. |
