# Supplementary material for: Screening and Prognostic Analysis of Immune-Related Genes in Pancreatic Cancer
Source: Front Genet. 2021 Oct 19;12:721419. doi: 10.3389/fgene.2021.721419 (PMC8560963; doi:10.3389/fgene.2021.721419)
Supplement: Supplementary file 1 [file DataSheet1.zip › Supplementary Tables/Table S6.docx]

**Table S6** Distribution of immune subtypes in pancreatic cancer samples with high immune infiltration score and pancreatic cancer samples with low immune infiltration score

| Group | Immune infiltration score | Group | Immune subtype | | | |
| --- | --- | --- | --- | --- | --- | --- |
|  |  |  | IS1 | IS2 | IS3 | IS4 |
| Hight-75% and low-25% | Stromal Score | Hight | 17 | 55 | 39 | 1 |
|  |  | Low | 27 | 2 | 1 | 7 |
|  | Immune Score | Hight | 18 | 54 | 40 | 0 |
|  |  | Low | 26 | 3 | 0 | 8 |
|  | Estimate Score | Hight | 16 | 56 | 40 | 0 |
|  |  | Low | 28 | 1 | 0 | 8 |
|  | Tumor Purity | Hight | 44 | 49 | 11 | 8 |
|  |  | Low | 0 | 8 | 29 | 0 |
| Hight-70% and low-30% | Stromal Score | Hight | 10 | 54 | 39 | 1 |
|  |  | Low | 34 | 3 | 1 | 7 |
|  | Immune Score | Hight | 13 | 51 | 40 | 0 |
|  |  | Low | 31 | 6 | 0 | 8 |
|  | Estimate Score | Hight | 12 | 53 | 39 | 0 |
|  |  | Low | 32 | 4 | 1 | 8 |
|  | Tumor Purity | Hight | 44 | 45 | 7 | 8 |
|  |  | Low | 0 | 12 | 33 | 0 |
