# Supplementary figures and images for: Screening and Prognostic Analysis of Immune-Related Genes in Pancreatic Cancer
Source: Front Genet. 2021 Oct 19;12:721419. doi: 10.3389/fgene.2021.721419 (PMC8560963; doi:10.3389/fgene.2021.721419)

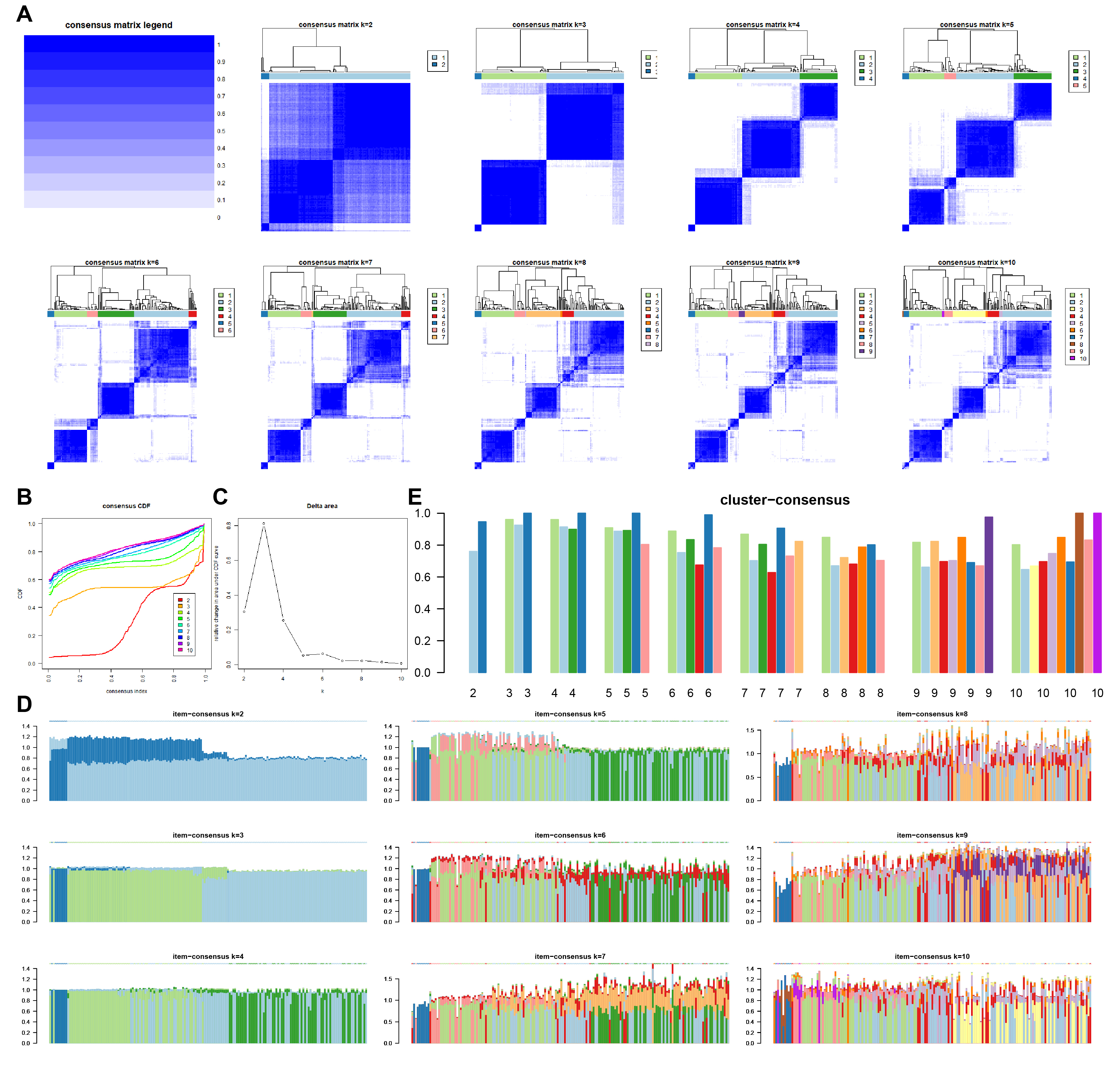

Supplement: Supplementary file 2 [file DataSheet2.zip › Supplementary Figures/Figure S1.jpg]

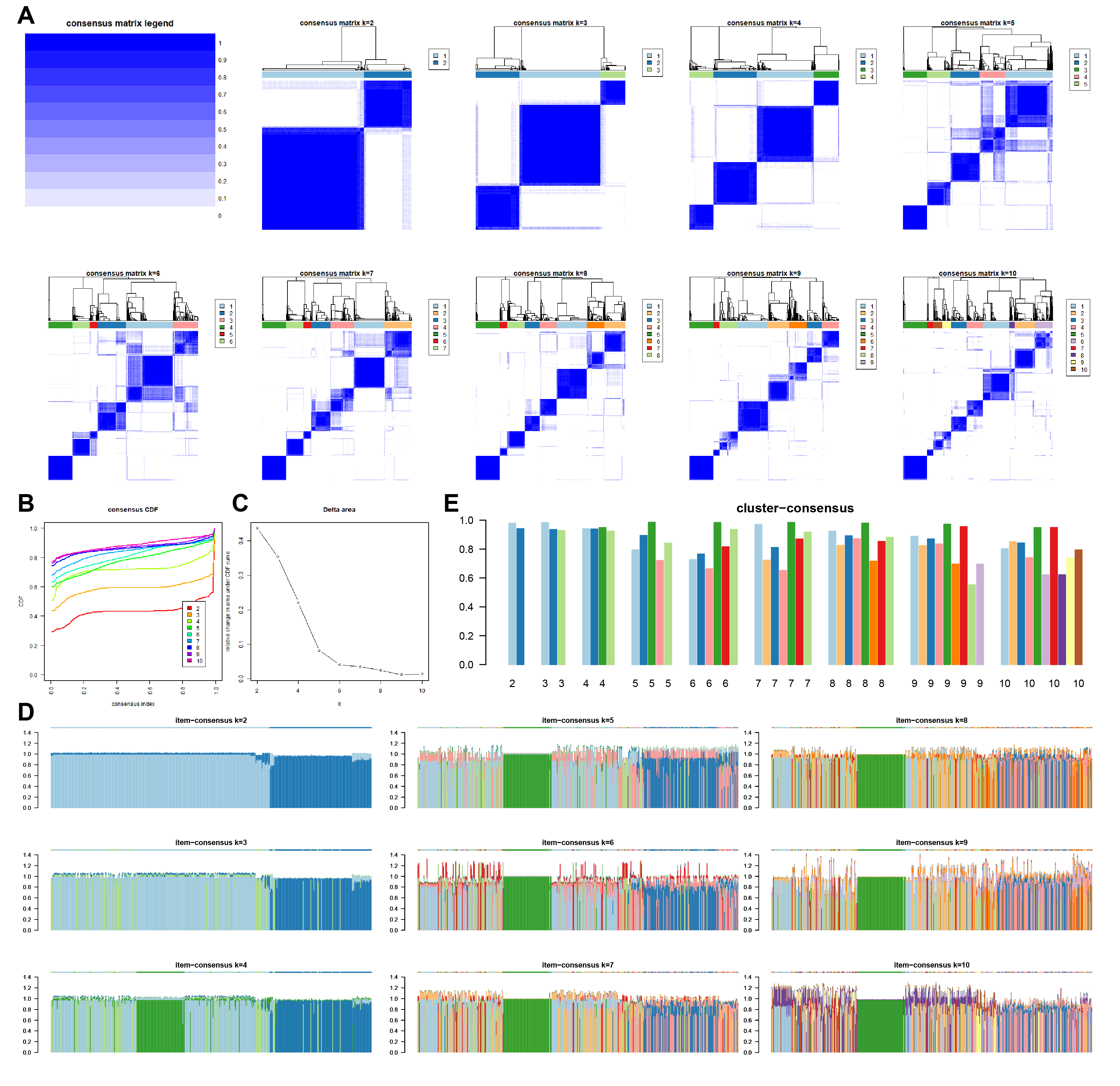

Supplement: Supplementary file 2 [file DataSheet2.zip › Supplementary Figures/Figure S2.jpg]

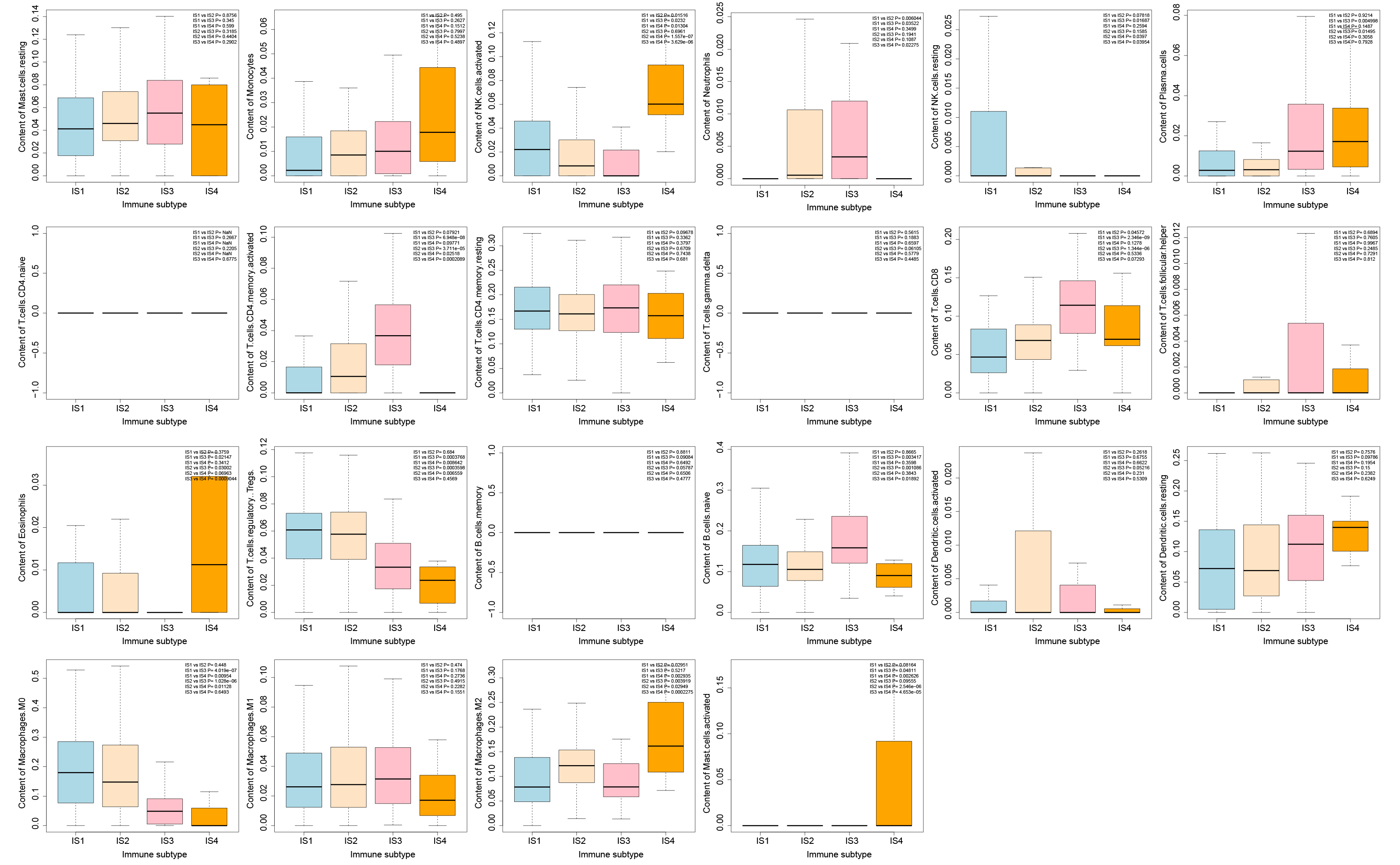

Supplement: Supplementary file 2 [file DataSheet2.zip › Supplementary Figures/Figure S3.jpg]

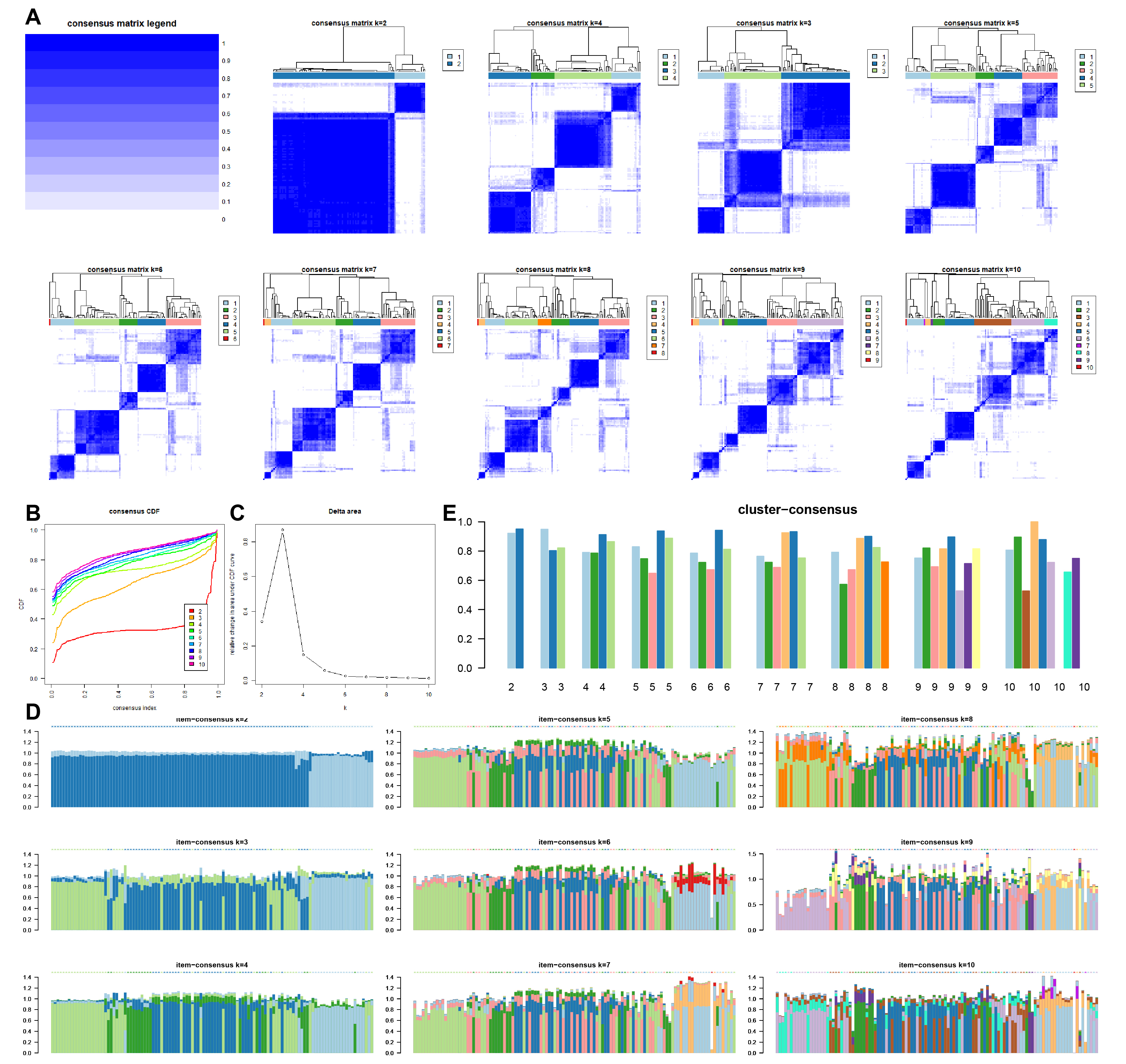

Supplement: Supplementary file 2 [file DataSheet2.zip › Supplementary Figures/Figure S4.jpg]
